# Supplementary material for: Altered microRNA Transcriptome in Cultured Human Liver Cells upon Infection with Ebola Virus
Source: Int J Mol Sci. 2021 Apr 6;22(7):3792. doi: 10.3390/ijms22073792 (PMC8038836; doi:10.3390/ijms22073792)
Supplement: Supplementary file 1 [file ijms-22-03792-s001.zip › Supplementary_File/D_ KEGG_Analysis_Results/16-30nt_pathway_Reston-96h-Huh7_vs_Control-96h-Huh7_down.mature_mirna_targets/hsa_pathwayResult.html]

| PathwayID | Definition | OriginalWebSite | Fisher-Pvalue | SelectionCounts | SelectionSize | Count | Size | FDR | Enrichment\_Score | GeneRatio | Genes |
| --- | --- | --- | --- | --- | --- | --- | --- | --- | --- | --- | --- |
| hsa04710 | Circadian rhythm - Homo sapiens (human) | http://www.genome.jp/kegg-bin/show\_pathway?hsa04710+8553+26224+5562+6500 | 3.628457e-03 | 4 | 164 | 31 | 7906 | 7.026004e-01 | 2.440278 | 0.024390 | BHLHE40//FBXL3//PRKAA1//SKP1 |
| hsa04918 | Thyroid hormone synthesis - Homo sapiens (human) | http://www.genome.jp/kegg-bin/show\_pathway?hsa04918+23439+84699+9586+493869+7849+7038 | 4.207188e-03 | 6 | 164 | 74 | 7906 | 7.026004e-01 | 2.376008 | 0.036585 | ATP1B4//CREB3L3//CREB5//GPX8//PAX8//TG |
| hsa04725 | Cholinergic synapse - Homo sapiens (human) | http://www.genome.jp/kegg-bin/show\_pathway?hsa04725+1128+1136+84699+9586+2775+9132+4893 | 8.524366e-03 | 7 | 164 | 112 | 7906 | 7.403868e-01 | 2.069338 | 0.042683 | CHRM1//CHRNA3//CREB3L3//CREB5//GNAO1//KCNQ4//NRAS |
| hsa04360 | Axon guidance - Homo sapiens (human) | http://www.genome.jp/kegg-bin/show\_pathway?hsa04360+1944+7976+4893+84612+5530+5532+6237+57715+90249 | 1.285937e-02 | 9 | 164 | 181 | 7906 | 7.403868e-01 | 1.890780 | 0.054878 | EFNA3//FZD3//NRAS//PARD6B//PPP3CA//PPP3CB//RRAS//SEMA4G//UNC5A |
| hsa05031 | Amphetamine addiction - Homo sapiens (human) | http://www.genome.jp/kegg-bin/show\_pathway?hsa05031+84699+9586+2904+5530+5532 | 1.317178e-02 | 5 | 164 | 68 | 7906 | 7.403868e-01 | 1.880356 | 0.030488 | CREB3L3//CREB5//GRIN2B//PPP3CA//PPP3CB |
| hsa04310 | Wnt signaling pathway - Homo sapiens (human) | http://www.genome.jp/kegg-bin/show\_pathway?hsa04310+1501+80319+2535+7976+5530+5532+6422+6500 | 1.802452e-02 | 8 | 164 | 160 | 7906 | 7.403868e-01 | 1.744136 | 0.048780 | CTNND2//CXXC4//FZD2//FZD3//PPP3CA//PPP3CB//SFRP1//SKP1 |
| hsa04728 | Dopaminergic synapse - Homo sapiens (human) | http://www.genome.jp/kegg-bin/show\_pathway?hsa04728+84699+9586+2775+2904+5530+5532+6323 | 1.898481e-02 | 7 | 164 | 131 | 7906 | 7.403868e-01 | 1.721594 | 0.042683 | CREB3L3//CREB5//GNAO1//GRIN2B//PPP3CA//PPP3CB//SCN1A |
| hsa05014 | Amyotrophic lateral sclerosis (ALS) - Homo sapiens (human) | http://www.genome.jp/kegg-bin/show\_pathway?hsa05014+2904+5530+5532+84134 | 2.094658e-02 | 4 | 164 | 51 | 7906 | 7.403868e-01 | 1.678887 | 0.024390 | GRIN2B//PPP3CA//PPP3CB//TOMM40L |
| hsa04022 | cGMP-PKG signaling pathway - Homo sapiens (human) | http://www.genome.jp/kegg-bin/show\_pathway?hsa04022+185+23439+84699+9586+4846+5530+5532+292 | 2.267697e-02 | 8 | 164 | 167 | 7906 | 7.403868e-01 | 1.644415 | 0.048780 | AGTR1//ATP1B4//CREB3L3//CREB5//NOS3//PPP3CA//PPP3CB//SLC25A5 |
| hsa04931 | Insulin resistance - Homo sapiens (human) | http://www.genome.jp/kegg-bin/show\_pathway?hsa04931+84699+9586+4846+8473+5562+10999 | 2.452654e-02 | 6 | 164 | 108 | 7906 | 7.403868e-01 | 1.610364 | 0.036585 | CREB3L3//CREB5//NOS3//OGT//PRKAA1//SLC27A4 |
| hsa00410 | beta-Alanine metabolism - Homo sapiens (human) | http://www.genome.jp/kegg-bin/show\_pathway?hsa00410+8639+1807+51733 | 3.033602e-02 | 3 | 164 | 33 | 7906 | 7.403868e-01 | 1.518041 | 0.018293 | AOC3//DPYS//UPB1 |
| hsa04151 | PI3K-Akt signaling pathway - Homo sapiens (human) | http://www.genome.jp/kegg-bin/show\_pathway?hsa04151+1027+1128+84699+9586+1944+3566+3678+8516+4846+4893+5562+7529+7534 | 3.219773e-02 | 13 | 164 | 354 | 7906 | 7.403868e-01 | 1.492175 | 0.079268 | CDKN1B//CHRM1//CREB3L3//CREB5//EFNA3//IL4R//ITGA5//ITGA8//NOS3//NRAS//PRKAA1//YWHAB//YWHAZ |
| hsa04370 | VEGF signaling pathway - Homo sapiens (human) | http://www.genome.jp/kegg-bin/show\_pathway?hsa04370+4846+4893+5530+5532 | 3.362190e-02 | 4 | 164 | 59 | 7906 | 7.403868e-01 | 1.473378 | 0.024390 | NOS3//NRAS//PPP3CA//PPP3CB |
| hsa05166 | Human T-cell leukemia virus 1 infection - Homo sapiens (human) | http://www.genome.jp/kegg-bin/show\_pathway?hsa05166+9184+84699+9586+2002+4893+5530+5532+292+7094 | 3.829094e-02 | 9 | 164 | 219 | 7906 | 7.403868e-01 | 1.416904 | 0.054878 | BUB3//CREB3L3//CREB5//ELK1//NRAS//PPP3CA//PPP3CB//SLC25A5//TLN1 |
| hsa04921 | Oxytocin signaling pathway - Homo sapiens (human) | http://www.genome.jp/kegg-bin/show\_pathway?hsa04921+2002+2775+4846+4893+5530+5532+5562 | 3.968922e-02 | 7 | 164 | 153 | 7906 | 7.403868e-01 | 1.401327 | 0.042683 | ELK1//GNAO1//NOS3//NRAS//PPP3CA//PPP3CB//PRKAA1 |
| hsa04110 | Cell cycle - Homo sapiens (human) | http://www.genome.jp/kegg-bin/show\_pathway?hsa04110+9184+1027+6500+7272+7529+7534 | 4.385109e-02 | 6 | 164 | 124 | 7906 | 7.403868e-01 | 1.358020 | 0.036585 | BUB3//CDKN1B//SKP1//TTK//YWHAB//YWHAZ |
| hsa04137 | Mitophagy - animal - Homo sapiens (human) | http://www.genome.jp/kegg-bin/show\_pathway?hsa04137+10241+3091+4893+6237 | 4.551809e-02 | 4 | 164 | 65 | 7906 | 7.403868e-01 | 1.341816 | 0.024390 | CALCOCO2//HIF1A//NRAS//RRAS |
| hsa04020 | Calcium signaling pathway - Homo sapiens (human) | http://www.genome.jp/kegg-bin/show\_pathway?hsa04020+185+1128+4846+22953+5530+5532+292+10345 | 4.730728e-02 | 8 | 164 | 193 | 7906 | 7.403868e-01 | 1.325072 | 0.048780 | AGTR1//CHRM1//NOS3//P2RX2//PPP3CA//PPP3CB//SLC25A5//TRDN |
| hsa04114 | Oocyte meiosis - Homo sapiens (human) | http://www.genome.jp/kegg-bin/show\_pathway?hsa04114+22849+5530+5532+6500+7529+7534 | 4.984407e-02 | 6 | 164 | 128 | 7906 | 7.403868e-01 | 1.302386 | 0.036585 | CPEB3//PPP3CA//PPP3CB//SKP1//YWHAB//YWHAZ |
| hsa04720 | Long-term potentiation - Homo sapiens (human) | http://www.genome.jp/kegg-bin/show\_pathway?hsa04720+2904+4893+5530+5532 | 4.994285e-02 | 4 | 164 | 67 | 7906 | 7.403868e-01 | 1.301527 | 0.024390 | GRIN2B//NRAS//PPP3CA//PPP3CB |
